# Progress in Medicine

**Published:** 1898-09-17

**Authors:** 


### PROGRESS IN MEDICINE.

### DISEASES OF THE CIRCULATORY SYSTEM.

(*Continued from page* 355.)

**Cardiac Hypertrophy in Arterio-sclerosis.**—Hasenfeld[6] comes to the following conclusions from an investigation into the subject. Arterio-sclerosis of mild or moderate degree, only microscopic, is quite common in the splanchnic arteries, but marked sclerotic changes are much less common than in the aorta, arteries of extremities, and the brain. Arterio-sclerosis only leads to hypertrophy of the left ventricle when the splanchnics or the thoracic aorta are severely affected. Arterio-sclerosis of the other vessels does not seem to have such an effect. In the five cases of contracted kidney examined all the chambers of the heart were hypertrophied. If at the same time there was marked sclerosis of the splanchnics the hypertrophy was most marked in the left ventricle. Should further examination confirm the results now given as regards the uniform hypertrophy of all parts of the heart, we must conclude that the cause of hypertrophy in contracted kidney increases the work of both sides of the heart, and excites it to increased activity.

**Effect of Pregnancy on the Heart.**—It is commonly believed that the heart undergoes hypertrophy during pregnancy, which, beginning in the early months, progresses right up to labour, after which the normal condition is rapidly restored. Vaquez and Millet,[7] however, consider that the evidences of hypertrophy will not stand scrutiny, because past observers took no account of intercurrent troubles, such as Bright's disease. The authors examined the hearts of a large number of pregnant women, but failed to detect hypertrophy, except where there was some collateral circumstance to account for it. Respiration is increased during pregnancy, both in depth and rate; and the dyspnœa induced by exertion during pregnancy is probably due to inability of the lung and pulmonary circulation to adapt themselves to the increased needs of the economy. The increased intra-pulmonary vascular tension is often shown by the accentuation of the pulmonary second sound. Given this condition of the pulmonary circulation, it is easy to understand that any over-exertion or weakness of the heart muscle will induce dilatation of the right ventricle.

This increased intra-pulmonary vascular tension is probably the cause of the malign influence of pregnancy on diseased hearts. It leads to engorgement of the heart capillaries and venules, and finally to their rupture, producing myocardial apoplexies. These hæmorrhages have their seat of election in the left auricle, and are most often associated with mitral stenosis. Now vascular tension is greatly increased during labour, and may most easily lead to multiple myocardial hæmorrhages and consequent collapse of the heart.

**Ulcerative Endocarditis.**—Washbourn[8] says that in cases of infective endocarditis there are two distinct conditions, firstly, the valvular incompetency caused by the mechanical condition of the valves, and, secondly, the septicæmia produced by the bacteria. Sometimes the mechanical, at other times the septic effects are the most marked; and in some cases there may be simply the symptoms of septicæmia without any evidence of cardiac disease. The symptoms due to the septic condition may be of various types. There may be a typhoid state with coma, collapse, high temperature, and profuse sweating; or there may be acute septicæmic symptoms with frequent rigors, irregular temperatures, sweating, and wasting; or the embolic processes may be most marked with symptoms similar to those of pyæmia and with acute aneurisms in various parts of the body.

The case must be treated either as a cardiac case or as one of septicæmia according to the symptoms. The only specific treatment is by serum. If streptococci are found in the blood one is sure of the cause, and antistreptococcic serum is often of the greatest value. But in some cases it does not seem to do much good, probably because the damage already done is so great. And the serum treatment cannot as yet be adopted in gonococcic endocarditis, as there is none as yet available for that purpose.

Powell[9] mentions some cases of infective endocarditis treated by yeast. In one case it was given by the mouth for several weeks with apparent benefit, but the patient refused to continue with the remedy, and finally died from the disease. In another case a fairly concentrated cultivation of yeast in saccharine water was injected into the subcutaneous tissues, with a successful result. In four other cases, however, no marked results followed, but they were in an advanced state with splenic and other embolisms. The yeast was given owing to the fact that the cells contain nuclein, and it is possible that nuclein is an indirect germicide by increasing the number of white corpuscles, on the constituents of which the germicidal property of blood serum depends.

Kerschensteiner[10] says that a malignant endocarditis arising in connection with pneumonia may be cured by (1) the pneumococcus; or (2) the streptococcus and staphyloccus. The different forms of the disease are differentiated by the course, temperature, curve and complications. When due to the pneumococcus the fever is continuous, whereas in the other forms it is intermittent. Infarcts and metastatic abscesses are very rare in pneumococcic endocarditis, but are usual when due to the other above-mentioned organisms. Pneumococcic endocarditis is not easily recognised when it is consecutive to acute pneumonia, and there is no meningitis. Pneumonia, endocarditis, and meningitis present a characteristic trio of lesions in pneumococcæmia.

**Intermittent Lameness.**—Charcot was the first to notice the occurrence of this condition in the human subject, though it has for some time been known in veterinary medicine. Bourgeois[11] has lately reinvestigated the subject. The affection consists of an intermitting painful paralysis, due apparently to arterial obliteration coming on suddenly, so that all motor power is suspended in arm or leg as the case may be. This arterial obliteration may be due primarily to atheroma, whether occurring in an arthritic subject or in connection with alcoholism, lead poisoning, malaria or senility, diabetes, or syphilis. The blood supply to the limbs is thus diminished. When, therefore, the patient is at rest there may be no inconvenience, but when any attempt at movement is made, particularly if an unaccustomed one be attempted, a sudden attack of pain, with loss of motor power, may result. Thus, whilst walking the patient may be suddenly affected with lameness; or the initial symptoms may be slow and insidious, walking, for instance, is accompanied by a certain amount of fatigue, and the more fatigued the patient becomes the more does he suffer from pain in one or both limbs; finally, the patient finds that after a few minutes walking he is obliged to sit down, unable to move another step. There is also added to the pain and inability to move a sense of extreme numbness, with sensations of burning and itching. These symptoms may become so marked that intolerable cramp with marked contracture may occur. These phenomena may be limited to the toes, but, as a rule, are more common in the calves and thighs, and even in the gluteal regions in certain cases. During an attack arterial pulsation is diminished, and may even be imperceptible. The reflex action is unaffected. The patient, when overcome by the severity of the symptoms, lies down to rest, when the majority of the painful symptoms disappear as if by magic. He may be able to resume walking, but there is soon a return of all the painful symptoms. In very many cases treatment is extremely satisfactory, for iodide of sodium, or potassium, with careful dieting have often completely relieved the patient. In the rare cases due to aneurism, the diagnosis followed by operation has completely relieved the disease.

6 Hasenfeld, Deut. Archiv. für Klin. Med. Bd. 59. 7 Vaquez and Millet, La Presse Médicale, Feb. 2, 1898. 8 Washbourn, Clin. Jour., June 29, 1898. 9 Powell, Brit. Med. Jour., April 9, 1898. 10 Kerschensteiner, Münch. Med. Woch., Aug. 3, 1898. 11 Bourgeois, Thèse de Paris, 1897.

